# Large-Scale Identification and Characterization of *Heterodera avenae* Putative Effectors Suppressing or Inducing Cell Death in *Nicotiana benthamiana*

**DOI:** 10.3389/fpls.2017.02062

**Published:** 2018-01-15

**Authors:** Changlong Chen, Yongpan Chen, Heng Jian, Dan Yang, Yiran Dai, Lingling Pan, Fengwei Shi, Shanshan Yang, Qian Liu

**Affiliations:** ^1^Key Laboratory of Pest Monitoring and Green Management, Ministry of Agriculture, Department of Plant Pathology, China Agricultural University, Beijing, China; ^2^Beijing Agro-Biotechnology Research Center, Beijing Academy of Agriculture and Forestry Sciences, Beijing, China; ^3^Qinzhou Entry-Exit Inspection and Quarantine Bureau, Guangxi, China; ^4^Central Political and Legal Affairs Commission of CPC Chengwu County Committee, Shandong, China

**Keywords:** *Heterodera avenae*, effector, suppression of plant defenses, BAX-triggered programmed cell death, *Nicotiana benthamiana*, PTI and ETI, interplay

## Abstract

*Heterodera avenae* is one of the most important plant pathogens and causes vast losses in cereal crops. As a sedentary endoparasitic nematode, *H. avenae* secretes effectors that modify plant defenses and promote its biotrophic infection of its hosts. However, the number of effectors involved in the interaction between *H. avenae* and host defenses remains unclear. Here, we report the identification of putative effectors in *H. avenae* that regulate plant defenses on a large scale. Our results showed that 78 of the 95 putative effectors suppressed programmed cell death (PCD) triggered by BAX and that 7 of the putative effectors themselves caused cell death in *Nicotiana benthamiana*. Among the cell-death-inducing effectors, three were found to be dependent on their specific domains to trigger cell death and to be expressed in esophageal gland cells by *in situ* hybridization. Ten candidate effectors that suppressed BAX-triggered PCD also suppressed PCD triggered by the elicitor PsojNIP and at least one R-protein/cognate effector pair, suggesting that they are active in suppressing both pattern-triggered immunity (PTI) and effector-triggered immunity (ETI). Notably, with the exception of isotig16060, these putative effectors could also suppress PCD triggered by cell-death-inducing effectors from *H. avenae*, indicating that those effectors may cooperate to promote nematode parasitism. Collectively, our results indicate that the majority of the tested effectors of *H. avenae* may play important roles in suppressing cell death induced by different elicitors in *N. benthamiana*.

## Introduction

*Heterodera avenae*, the most commonly reported species of cereal cyst nematode (CCN), causes substantial crop yield losses of 30–100% in wheat production worldwide (Bonfil et al., 2004; Nicol et al., 2007). The infective second-stage juveniles (J2s) of CCN invade the lateral roots or root tips of plants and migrate intracellularly toward the vascular cylinder, where they form feeding sites called syncytia. The CCN becomes sedentary and remains associated with the developing syncytium from which it obtains nutrients necessary for growth and development (Sobezak and Golinowski, 2009). A better understanding of the mechanisms underlying the interactions between CCN and its hosts is important for the development of new control strategies.

Plants respond to infection using two modes of innate immunity (Jones and Dangl, 2006; Tsuda and Katagiri, 2010). The first mode of immunity is referred to as “pattern-triggered immunity” (PTI) and is triggered by microbe-associated or pathogen-associated molecular patterns (MAMPs or PAMPs). Through evolution, adapted pathogens secrete effector proteins into plant cells and suppress PTI (Boller and He, 2009). To counter pathogens, plants have evolved resistance (R) proteins that specifically recognize certain pathogen effectors, resulting in initiation of the second mode of plant immunity, which is referred to as “effector-triggered immunity” (ETI) (Chisholm et al., 2006). The dynamic co-evolution of plants and pathogens is ongoing, and some pathogens have acquired effectors that interfere with ETI (Jones and Dangl, 2006). Thus, plants and microbial pathogens are engaged in an endless “arms race.”

Plant pathogens secrete proteins and other molecules known as effectors that modulate plant defenses and permit the pathogens to colonize plant tissue (Hogenhout et al., 2009). Systematic identification and characterization of the effectors that regulate plant immunity has been reported for various pathogens. Agro-infiltration in tobacco is the most popular assay used in the identification of genes regulating plant immunity because it is simple and quick. In bacteria, *Pseudomonas syringae* pv. *tomato* DC3000 expresses 36 bacterial type III effectors, 32 of 35 tested effectors can suppress HopA1-dependent ETI in tobacco, and many effectors can also suppress PTI (Guo et al., 2009). In oomycetes, the avirulence homolog (Avh) proteins share RXLR-dEER motifs, rendering them all candidate effectors. Of 169 effectors tested, most of the Avh proteins identified in the *Phytophthora sojae* genome suppress BAX-triggered programmed cell death (BT-PCD) using an *Agrobacterium tumefaciens-*mediated transient expression assay in *Nicotiana benthamiana* (Wang et al., 2011). In the fungus *Ustilaginoidea virens*, more than half of 30 randomly selected putative effectors identified in the genome were found to suppress the *Burkholderia glumae*-triggered hypersensitive reaction (HR) in *N. benthamiana* (Zhang et al., 2014). In addition to functioning as virulence factors that cripple host defenses, some effectors have been demonstrated to trigger host immunity. Eleven of 169 effectors in *P. sojae* were shown to trigger cell death, chlorosis, or mottling in *N. benthamiana* leaves (Wang et al., 2011). Among 42 *Magnaporthe oryzae* effectors identified in infected rice leaves, five proteins induced cell death in rice protoplasts only when they contained a signal peptide (SP) (Chen et al., 2013). Eight of 119 putative effectors from *U. virens* were proven to trigger cell death in rice protoplasts, and the SP of these proteins are essential for their cell-death-inducing activity (Fang et al., 2016). Recently, bioinformatic analyses of the draft genome sequences and transcriptome in plant parasitic nematodes have identified a lot of candidate effector proteins (Abad et al., 2008; Opperman et al., 2008; Thorpe et al., 2014; Eves-van den Akker et al., 2016; Li et al., 2016), which provide resources for large scale identification of nematodes effectors with the ability to suppress or induce plant defenses.

Unlike bacterial and oomycete effectors, a limited number of plant parasitic nematode (PPN) effectors have been functionally characterized. Some nematode effectors have been found to suppress host immunity through various molecular mechanisms (Haegeman et al., 2012; Favery et al., 2016). Overexpression of *Meloidogyne incognita* calreticulin in *Arabidopsis thaliana* suppresses the induction of defense marker genes and callose deposition after treatment of the plant with the PAMP elf18 (Jaouannet et al., 2013). Mj-FAR-1, a fatty acid- and retinol-binding protein secreted by *M. javanica*, has been shown to interfere with host lipid-based defenses and thereby facilitate infection (Iberkleid et al., 2013). MjTTL, a transthyretin-like homolog from *M. javanica*, exploits the host’s ferredoxin: thioredoxin system and drastically increases host reactive oxygen species-scavenging activity, resulting in suppression of plant basal defenses (Lin et al., 2016). The *M. incognita* putative secretory protein MiMsp40 suppresses programmed cell death (PCD) triggered by BAX, MAPK cascades and the ETI elicitors R3a/Avr3a, and overexpression of *MiMsp40* in plants suppresses the deposition of callose and the expression of PTI marker genes (Niu et al., 2016). The CEP12 peptide from *Globodera rostochiensis* suppresses resistance-gene-mediated cell death, thereby suppressing plant immunity (Chronis et al., 2013). Several members of the SPRYSEC effector family in *G. rostochiensis* function as selective suppressors of defense-related PCD (Diaz-Granados et al., 2016). VAPs from *G. rostochiensis* and *H. schachtii* only affected the programmed cell death mediated by surface-localized immune receptors (Lozano-Torres et al., 2014). HgGLAND18 from *H. glycines* strongly suppresses both basal and hypersensitive cell death innate immune responses, and immunosuppression requires the presence and coordination between multiple protein domains (Noon et al., 2016). Annexin from *H. avenae* can suppress PCD triggered by BAX and the induction of marker genes of PTI in *N. benthamiana* (Chen et al., 2015). Moreover, effectors that induce plant defenses have also been described in nematodes. GrEXPB2 inhibits the cell death induced by PiNPP, AtRX and AvrBs2/Bs2 in tobacco leaves, and also induces chlorosis in *N. benthamiana* and cell death in tomato and potato. GrEXPB2 may have the dual properties of suppressing and eliciting plant defenses (Ali et al., 2015). Cg1 from *M. javanica* appears to be involved in triggering the immune response in host plants carrying the *Mi-1* resistance gene (Gleason et al., 2008). The effector protein RBP-1 of *G. pallida* is reported to elicit cell death through the NB-LRR protein Gpa2 (Sacco et al., 2009). Transient expression of the *G. rostochiensis* effector VAP1 in tomato plants harboring *Cf-2* and *Rcr3^pim^* triggers a defense-related PCD in plant cells (Lozano-Torres et al., 2012). HaEXPB2, a predicted expansin-like protein from *H. avenae*, causes cell death when expressed with the SP in *N. benthamiana* (Liu et al., 2016). However, neither large-scale identification of nematode effectors that regulate plant immunity nor cooperation between effectors has been reported to date.

RNA silencing is a major type of defense mechanism against RNA viruses (Ding, 2010). Viral infection induces host siRNAs that direct the cleavage of viral RNAs. To counter this type of defense, viruses have developed suppressors of viral RNA silencing that interfere with the RNA silencing machinery and promote infection (Incarbone and Dunoyer, 2013). However, viruses are not the only organisms that are able to promote infection by blocking RNA silencing. Three known bacterial effectors can suppress the miRNA pathway in *A. thaliana* (Navarro et al., 2008). Two oomycete RxLR effectors, PSR1 and PSR2, of *P. sojae* were shown to suppress RNA silencing in plants by inhibiting the biogenesis of small RNAs (Qiao et al., 2013). A PSR2-like effector from the related species *P. infestans* can also suppress RNA silencing (Xiong et al., 2014). Recently, it has also been reported that transgenic tobacco plants expressing a known viral suppressor display increased susceptibility to root-knot nematode (RKN) infection and that RKN parasitism may suppress host RNA silencing (Walsh et al., 2017). To date, however, no suppressors of RNA silencing have been reported in nematodes.

As a biotrophic pathogen, *H. avenae* needs to suppress plant defenses during the entire parasitic process. How many effectors of *H. avenae* are involved in suppressing plant immunity? What is the interplay between the effectors? Recently, the transcriptomes of *H. avenae* during infection of wheat and incompatible hosts have been published (Kumar et al., 2014; Zheng et al., 2015; Yang et al., 2017), thereby providing a resource for the large-scale mining of effectors in *H. avenae*. In this study, we systematically investigated the ability of a large number of putative effectors encoded in the *H. avenae* transcriptome to suppress plant defenses. The results revealed that most of the putative effectors had the potential to suppress PCD triggered by BAX, effectors or elicitin. Interestingly, several of the putative effectors could trigger cell death in *N. benthamiana* by themselves. We also explored the interplay between effectors that suppress PCD and those that induce PCD. Our results provide important information to understand how nematodes regulate plant defenses.

## Materials and Methods

### Nematodes and Plants

*Heterodera avenae* was propagated on wheat (*Triticum aestivum* cv. Aikang 58) in an artificial environment. Six different life stages of the nematodes (egg, preJ2, postJ2, J3, J4 and adult female) were obtained as previously described (Chen et al., 2015).

*Nicotiana benthamiana* plants were grown in a growth room for 4–6 weeks at approximately 25°C with a 14 h light/10 h dark cycle.

### Developmental Expression Analysis

Analysis of the expression of specific genes during development was conducted as previously described (Chen et al., 2015). The primers used to detect the expression of these genes and of the reference gene *GAPDH-1* are listed in Supplementary Table S1.

The expression cluster heatmap tools available on BMKCloud^1^ were used to construct a heatmap of the developmental expression of different genes. The developmental expression trends of genes that suppress BT-PCD and those that induce PCD were also analyzed separately using these tools.

### Cell-Death Suppression Assay in *N. benthamiana*

The ORF sequences of candidate effector genes of *H. avenae* and *eGFP* were constructed into the Potato virus X (PVX) vector pGR107 (Jones et al., 1999) with a flag-tag fused at the N-terminus using the In-Fusion HD Cloning Kit (Clontech, United States) as described in the user manual or by the method of digestion and connection. The necrosis elicitor gene *psojNIP* (Dinah et al., 2002) was constructed into the pGR107 vector with an HA-tag fused at the C-terminus using the In-Fusion HD Cloning Kit (Clontech, United States). The primers used for vector construction are listed in Supplementary Table S1. These constructs were confirmed by sequencing and transformed into *A. tumefaciens* strain GV3101 for infiltration. By using the PVX vectors, the virus spreads systemically and leads to massive overexpression of the effector proteins in *N. benthamiana*. In addition, the recombinant construct of pGR107-Bax and the constructed vector PMD1 (Tai et al., 1999) expressing Avr3a, R3a, Rbp-1 or Gpa2 were kindly provided by other researchers (see Acknowledgments).

Assays of the suppression of BAX-, psojNIP-, Avr3a/R3a-, and Rbp-1/Gpa2-triggered PCD were performed as previously described (Wang et al., 2011) except that *A. tumefaciens* cells carrying the elicitor genes were infiltrated only at 24 h after the initial inoculation. The assays were independently repeated 2–3 times, with 3–6 tobacco plant replicates inoculated on three leaves of each plant each time. Photographs of the infiltrated leaves of *N. benthamiana* were obtained approximately 7 days after infiltration directly or after decolorization of the leaves by boiling in 95% ethanol for 20 min. The degree of PCD of leaves treated with vectors carrying the candidate genes and control genes followed by necrosis elicitor genes, referred to as the “necrosis index,” was scored on a ten-point scale according to the size of the necrotic area (grade 1 for 10% necrosis of the whole circle area, grade 2 for 20%, and so on) (Chen et al., 2015). The necrosis percentage relative to the *eGFP* of each gene was calculated by comparing the necrosis index of each gene followed by BAX to that of the eGFP control.

For verification of gene expression, western blotting was performed. Proteins were extracted from the infiltrated portions of leaves of *N. benthamiana* using the Plant Protein Extraction Kit (CoWin Biosciences, China). Anti-BAX antibody, anti-HA antibody, anti-FLAG antibody, anti-HIS antibody, or anti-β-Actin antibody, and DAB Kit (CoWin Biosciences, China), BCIP/NBT Kit (CoWin Biosciences, China) or EasySee Western Blot Kit (TransGen Biotech, China) for color visualization were used to detect the expression of proteins carrying BAX, HA, FLAG or HIS tags, or the protein of β-Actin.

### Assay of Cell Death Induction by Three Candidate Effectors without Structural Domains by Transient Expression in *N. benthamiana*

The ORF sequences (without SPs) of *isotig12969*, *isotig19390* and *isotig16511* and sequences of fragments of these genes with the structural domains deleted were obtained by direct PCR or overlap PCR and constructed into the pND108 vector (Zhang et al., 2005) with 6 × His tag. The infiltration procedures and verification of protein expression were the same as those described in the subsection titled “Cell-death suppression assay in *N. benthamiana*” except that no elicitors were infiltrated. The assays were independently repeated three times, with 3–4 plant replicates inoculated on 2–3 leaves of each plant each time. The average necrosis index obtained in the three determinations was calculated.

### Assay of Cooperation among *H. avenae* Candidate Effectors by Transient Expression in *N. benthamiana*

Four candidate effector genes (*isotig16511*, *isotig16978*, *isotig19390,* and *isotig12969*) triggering cell death in *N. benthamiana* leaves were constructed into the vector pND108 with a His tag; 10 additional genes were constructed into the pGR107 vector with a 3× flag tag and were tested for their ability to suppress cell death induced by the former four genes. The experiment was performed as described in the subsection titled “Cell-death suppression assay in *N. benthamiana.*”

### Validation of the SP Secretion Activity of Candidate Effector Genes

The SP secretion activity of the candidate effector genes was tested using a yeast secretion assay as previously reported (Oh et al., 2009) with some modifications. The ORF sequences (including the predicted SP coding sequences and the following gene sequences without termination codon sequences) were amplified using the primers listed in Supplementary Table S1 and constructed into pSUC2 using the In-Fusion HD Cloning Kit (Clontech, United States). The constructed plasmid was transformed into the invertase-negative yeast strain YTK12 according to the instructions provided with the Frozen-EZ Yeast Transformation II kit (Zymo Research, United States). The transformants were then assayed for secretion activity as previously described (Oh et al., 2009).

### mRNA *in Situ* Hybridization in Nematodes

Digoxigenin (DIG)-labeled sense (control) and antisense cDNA probes were synthesized by asymmetric PCR (Huang et al., 2003) using the primers listed in Supplementary Table S1. *In situ* hybridization was conducted as previously described (Chen et al., 2015) but with the hybridization temperature adjusted according to the probes used.

### Systemic Transient Expression of *H. avenae* Putative Effectors in *N. benthamiana*

*Agrobacterium tumefaciens* GV3101 strains carrying the tested 67 candidate effector genes and the *eGFP* control were constructed as described in the subsection “Cell-death suppression assay in *N. benthamiana.*” Freshly cultured *A. tumefaciens* cells were collected and resuspended in infiltration buffer (10 mM MgCl_2_, 10 mM 2-(N-morpholino) ethanesulfonic acid, 4-morpholineethanesulfonic acid (MES, pH 5.6) and 200 μM acetosyringone) to a final OD_600_ of 0.5 and incubated at room temperature for 3 h. For the infiltration, 2/3 of the area of one leaf was infiltrated. Each gene was assayed on five plants with 1–2 leaves for each plant with *eGFP* as a negative control. Photographs of the infiltrated *N. benthamiana* leaves were obtained approximately 7 days after infiltration. The assay was repeated independently at least three times.

### Screening of RNA Silencing Suppressors in *H. avenae*

The ORF sequences of 54 candidate *H. avenae* effector genes, *P19* and *eGFP* were constructed into the pGD binary vector harboring 35S promoter (Goodin et al., 2002) using the In-Fusion HD Cloning Kit (Clontech, United States) and the primers listed in Supplementary Table S1. The recombinant constructs were transformed into the *A. tumefaciens* GV3101 strains for infiltration. Freshly cultured *A. tumefaciens* cells carrying the recombinant vectors were inoculated at 1:100 into LB liquid medium containing 100 mg/L kanamycin, 2 mg/L tetracycline, 10 mM MES and 20 μM acetosyringone and cultured to OD_600_ = 1.0 by shaking at 28°C. The *A. tumefaciens* cells were then collected by centrifugation at 5000 rpm for 5 min and resuspended in infiltration buffer (10 mM MgCl_2_, 10 mM MES and 150 μM acetosyringone). Leaves of 4-week-old *N*. *benthamiana* plants were co-infiltrated with mixed *Agrobacterium* cultures harboring sense GFP (sGFP) expression plasmid with different combinations of empty vector, *P19*, and each candidate effector (Zhang et al., 2017). The empty pGD vector and pGD-p19 were used as the negative and positive controls, respectively. Each treatment or control was assayed on five plants with 1–2 leaves for each plant. The phenotypes of infiltrated *N. benthamiana* were visualized under a UV lamp 3 days later, and photographs were taken using an orange filter. The assay was repeated at least three times independently.

### Statistical and Bioinformatic Analyses

One-way ANOVA (Duncan test) or independent-samples t-tests conducted in SPSS 13.0 were used to analyze differences between different samples. Structural domain prediction of the genes was conducted by SMART^2^.

## Results

### Large Numbers of Candidate Effectors from *H. avenae* Can Suppress BT-PCD

In our previous study, we have sequenced transcriptomes of the pre- and post-parasitic stages of *H. avenae* (Yang et al., 2017). The following criteria were used to mine putative effectors from the transcriptomic data: transcripts of length less than 1000 bp lacking a transmembrane domain sequence; transcripts homologous to reported effectors from plant or animal parasitic nematodes (Blastx *e*-value < 1e^-6^, Similarity > 50%); transcripts specifically highly expressed at parasitic stages (FDR < 0.001, |log_2_ (fold change)| > 1). Approximately 300 candidate effectors were identified after the bioinformatics analysis. We randomly selected ∼30% of these, i.e., 95 candidate effectors (GenBank accession numbers listed in Supplementary Table S2) and evaluated their plant defenses suppression abilities in this research. According to the gene annotation, a lot of candidate genes hit previously reported effectors, such as some gland proteins, vap, 14-3-3, expansin and cellulase.

**FIGURE 1:**
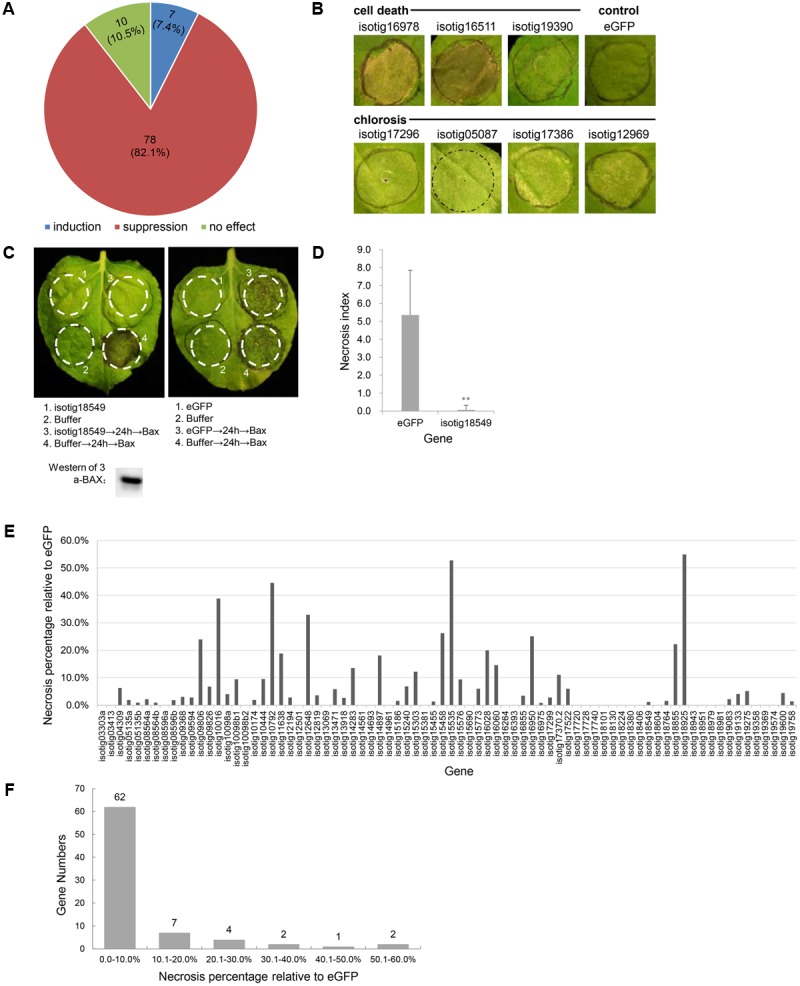
Effect of *Heterodera avenae* candidate effectors on *Nicotiana benthamiana* PCD. **(A)** Number and proportion of putative effector genes that induce PCD, suppress BAX-triggered cell death (BT-PCD) or have no effect on leaves of *N. benthamiana*. **(B)** Putative effectors that trigger cell death and chlorosis symptoms in *N. benthamiana* compared to eGFP as the negative control. **(C)** Suppression of BT-PCD in *N. benthamiana* by effectors (example isotig18549). *N. benthamiana* leaves were infiltrated with buffer or with *Agrobacterium tumefaciens* cells carrying *isotig18549* or the negative control *eGFP* gene; infiltration was either performed alone or followed 24 h later by infiltration with *A. tumefaciens* cells carrying a mouse *Bax* gene. Western blotting confirmed the expression of BAX. **(D)** Necrosis indices of the infiltration spots of the example gene *isotig18549* and control *eGFP* followed by *Bax*. Each column shows the mean and standard deviation. The columns with asterisks show a statistically significant reduction of the necrosis index of isotig18549 compared with that of eGFP (*P* < 0.01). **(E)** The necrosis percentage relative to eGFP of each of the 78 BT-PCD suppressing effector candidates. The necrosis percentage was calculated by comparing the necrosis index of each gene followed by Bax to that of the eGFP control. **(F)** Distribution of the necrosis percentage relative to eGFP of the 78 BT-PCD-suppressing effector candidates.

Because the BT-PCD suppression assay has been proven to be a valuable initial screening tool for pathogen effectors capable of suppressing defense-associated PCD (Abramovitch et al., 2003; Dou et al., 2008), we used this method to identify *H. avenae* candidate effectors. *N. benthamiana* leaves were infiltrated with *Agrobacterium* strains carrying each gene 24 h prior to infiltration of the *BAX*-carrying strain, and the resulting necrosis index was scored and compared with that of a negative *eGFP* control. The results showed that, of the 95 candidate effector genes evaluated, 78 genes (82.1%) suppressed BT-PCD to variable degrees; 10 genes (10.5%) had no obvious effect on BT-PCD, and 7 genes (7.4%) induced cell death or chlorosis in *N. benthamiana* leaves (**Figures 1A,B**).

As an example of a representative suppression gene, we display the results obtained for the gene *isotig18549* (**Figures 1C,D**). The leaf infiltration spot produced by isotig18549 followed by BAX was not as necrotic as that produced by eGFP followed by BAX (**Figure 1C**). Western blotting was conducted to verify the expression of BAX of the leaf spot of infiltration of isotig18549 followed by BAX from the translational level. Furthermore, quantitative comparison showed that the necrosis index of isotig18549 (0.1) followed by BAX was much lower than that of the eGFP control (5.4). This finding suggests that isotig18549 can suppress BT-PCD to some extent. Similarly, other genes that yielded necrosis indices lower than that produced by the eGFP control when used to infiltrate leaves followed by BAX were all considered to be BT-PCD-suppressive. However, these genes exhibited variable suppression of necrosis relative to the eGFP control (**Figure 1E**); 62 (79.5%) of the tested genes had high suppression activity, with relative necrosis rates lower than 10% (**Figure 1F**).

### Expression Characteristics of Candidate Effectors Regulating Plant Defenses

**FIGURE 2:**
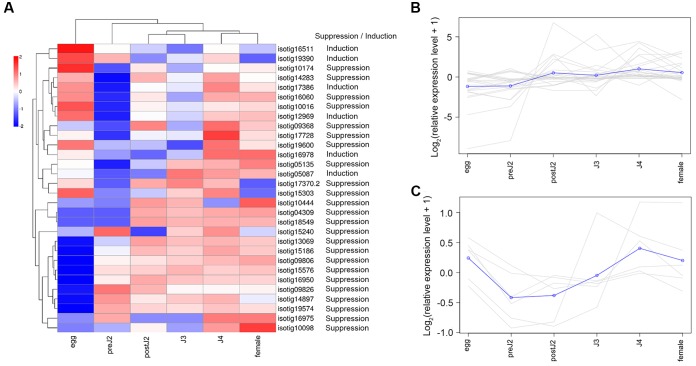
Developmental expression pattern of *Heterodera avenae* candidate effector genes. **(A)** Heatmap of the expression of all the selected candidate effector genes. The common expression patterns of selected BT-PCD-suppressing effector genes and PCD-inducing effector genes are shown in **(B,C)**, respectively.

We used qRT-PCR to analyze the expression of 30 genes (including 24 defense-suppressing genes and 6 defense-inducing genes) at various developmental stages. The expression patterns of these genes were then further analyzed using the expression cluster heatmap tools available on BMKCloud^3^. The heatmap of the developmental expression of different genes shows that although these genes displayed diverse transcription patterns during parasitism, on the whole most genes were more highly expressed during post-parasitic stages (parasitic second-, third- and fourth-stage juvenile (postJ2, J3, and J4) and female) (**Figure 2A**). In addition, genes suppressing BT-PCD (**Figure 2B**) and those inducing PCD (**Figure 2C**) displayed different expression pattern trends; that is, the latter were less active during the pre-parasitic second-stage and early parasitic stage juvenile (preJ2-J3).

**FIGURE 3:**
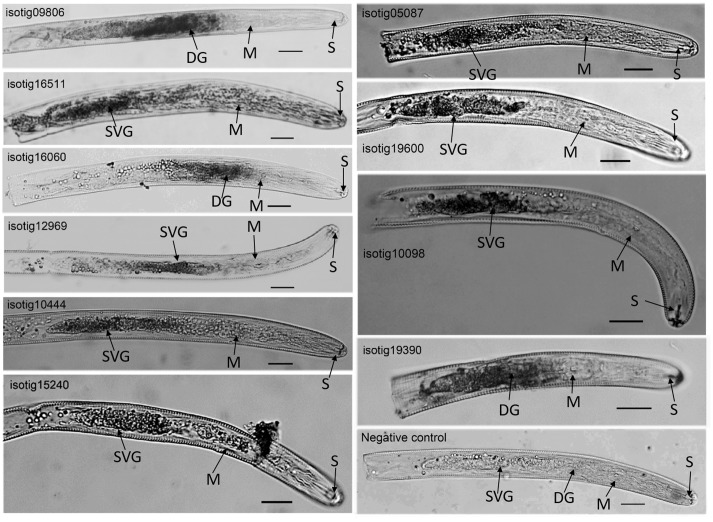
*In situ* hybridization of selected *Heterodera avenae* candidate effector genes in preJ2s. The signal of antisense DIG-labeled cDNA probes is localized within the gland cells, with sense probes as the negative control. DG, dorsal gland cell; SVG, subventral gland cell; M, metacorpus; S, stylet. Scale bar = 20 μm.

Localization of the expression of ten candidate effector genes, including four genes (*isotig12969* (hit profilin-1 [*Ascaris suum*]), *isotig19390* (hit profilin [*Brugia malayi*]), *isotig16511* (hit endonuclease G [*A. suum*]) and *isotig*05087 (hit ShTK domain containing protein [*B. malayi*])) that could trigger PCD in *N. benthamiana* and six genes (*isotig09806* (hit flavin-binding monooxygenase-like protein [*Necator americanus*]), *isotig10444* (hit zinc metalloproteinase nas-10 [*A. suum*]), *isotig15240* (hit melibiase family protein [*B. malayi*]), 19600 (hit phospholipase a2-like protein [*A. suum*]), 10098 (hit acid phosphatase [*H. avenae*]) and *isotig16060* (hit 14-3-3 protein [*H. glycines*])) that could suppress BT-PCD, was accomplished using *in situ* hybridization. Transcripts of all ten genes were observed in gland cells in the preJ2 stage of *H. avenae* (**Figure 3**), demonstrating that the products of these genes could act as secreted effectors. With the exception of three genes *isotig09806*, *isotig16060* and *isotig19390* expressed in dorsal gland cells transcriptionally, the other seven genes were all expressed in subventral gland cells by *in situ* hybridization. No signal was detected in negative controls in which sense probes were used (**Figure 3**).

### Functional Validation of Predicted SPs of Candidate Effectors

**FIGURE 4:**
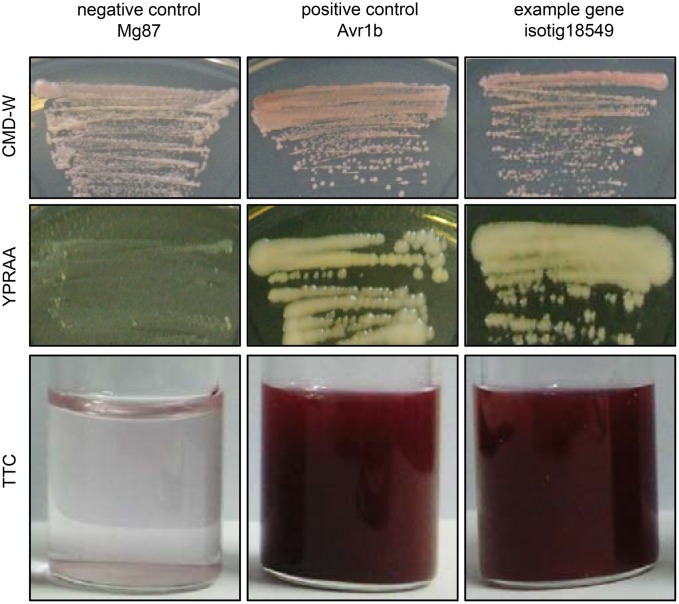
Functional validation of the signal peptides of *Heterodera avenae* candidate effector genes (example *isotig18549*). In the yeast invertase secretion assay, yeast YTK12 strains containing the pSUC2 vector bearing the SP fragments fused in-frame to the invertase gene were able to grow in both CMD-W and YPRAA media and to reduce TTC to red formazan, implying secretion of invertase. The SPs of *Phytophthora sojae* Avr1b and *Magnaporthe oryzae* Mg87 served as positive and negative controls, respectively.

To identify the secretory activity of the predicted SPs of some candidate effectors, we adopted a yeast secretion system (Jacobs et al., 1997; Fang et al., 2016). In this system, pSUC2 vectors containing the predicted SP nucleotide sequence of each gene fused with the truncated *SUC2* gene (encoding invertase) lacking its own SP were constructed. When the fusion constructs were transformed into the invertase-secretion-deficient yeast strain YTK12, the invertase with fused SP was secreted into the medium, where it degraded the sole carbon source raffinose into simple sugars, permitting survival of the YTK12 cells (**Figure 4**). The secretion of invertase was verified using an enzymatic activity test based on reduction of the dye 2,3,5-triphenyltetrazolium chloride (TTC) to the insoluble red-colored compound triphenylformazan (**Figure 4**). In this experiment, 33 of the 53 tested SPs were shown to have secretion activity (Supplementary Table S2). A typical result is shown in **Figure 4**, in which the results obtained for isotig18549 are presented along with those obtained using the SPs of *P. sojae* Avr1b and *M. oryzae* Mg87, which served as positive and negative controls, respectively (Gu et al., 2011; Fang et al., 2016).

### Systemic Transient Expression of *H. avenae* Candidate Effectors in *N. benthamiana*

**FIGURE 5:**
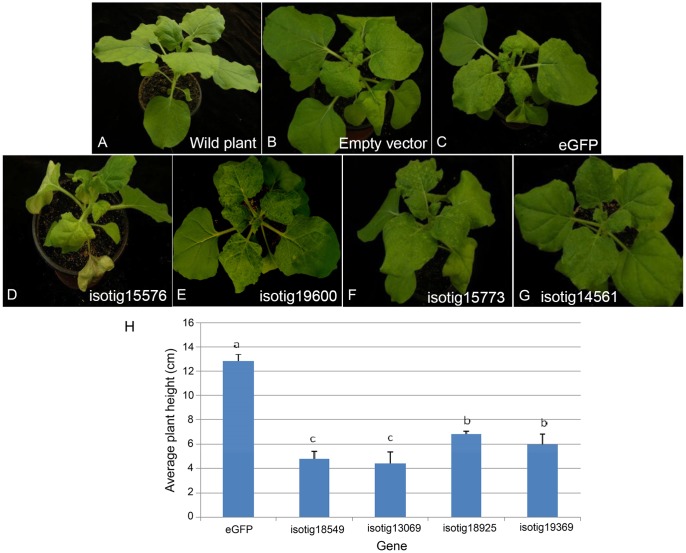
Symptoms of systemic transient expression of *Heterodera avenae* effectors in *Nicotiana benthamiana*. **(A)** Untreated wild plant. **(B)** Empty vector control. **(C)** eGFP control. **(D)** Severe necrosis with wilting and even withering (example isotig15576). **(E)** Moderate necrosis (example isotig19600). **(F)** Aggravation of PVX symptoms (example isotig15773). **(G)** No obvious difference compared to the eGFP control (example isotig14561). **(H)** Stunting indicated by a significant decrease in average plant height after infiltration with isotig18549, isotig13069, isotig18925, or isotig19369 compared to the eGFP control (*P* < 0.05).

Systemic transient expression of exogenous genes in *N. benthamiana* can be used to determine whether the genes play roles in plant infection. PVX vector can infect *N. benthamiana* systematically, transmit and accumulate acropetally and has the ability to express proteins rapidly and massively. We thus used a system mediated by *A. tumefaciens* in which the PVX vector was used to express 67 putative effector candidates in *N. benthamiana.* The results (Supplementary Table S2) showed that 5 genes induced severe necrosis, producing wilting and even withering (**Figure 5D**), 13 genes induced moderate necrosis in which the plants displayed some necrotic spots (**Figure 5E**), and 4 genes induced plant stunting, resulting in significantly smaller plant height than that of the *eGFP* control (*P* < 0.05) (**Figure 5H**). Half of the genes tested (34 genes) aggravated the PVX symptoms; that is, these genes caused the leaves to show more severe mosaic, chlorotic and mottled symptoms compared to the *eGFP* and empty vector controls, but no necrosis appeared (**Figure 5F**). The remaining 11 genes caused no obvious differences compared to the empty vector and *eGFP* controls (**Figure 5G**).

### None of the Effectors Tested Serve As RNA-Silencing Suppressors

**FIGURE 6:**
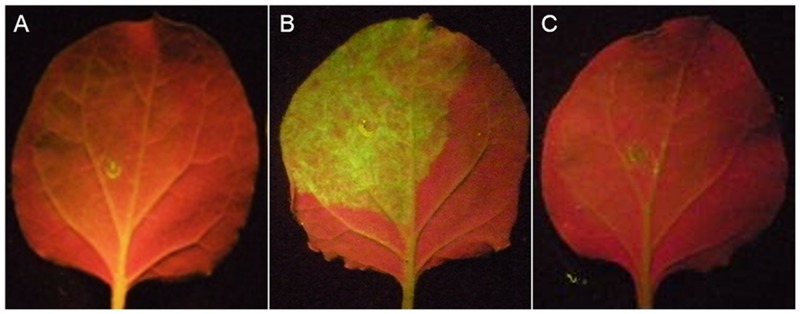
RNA-silencing suppression assay of candidate *Heterodera avenae* effectors in *Nicotiana benthamiana* (example isotig18549). **(A)** Negative control: *N. benthamiana* leaves were infiltrated with a mixture of *Agrobacterium tumefaciens* cells containing the empty pGD vector and pGD-eGFP showing no green fluorescence. **(B)** Positive control: *N. benthamiana* leaves were infiltrated with a mixture of *A. tumefaciens* cells containing pGD-p19 and pGD-eGFP showing green fluorescence. **(C)** Example isotig18549: *N. benthamiana* leaves were infiltrated with a mixture of *A. tumefaciens* cells containing pGD-isotig18549 and pGD-eGFP showing no green fluorescence.

Individual effector and *GFP* genes were coexpressed by *A. tumefaciens* infiltration in the leaves of *N. benthamiana*. *GFP* genes are silenced by siRNAs induced by the infiltrated *GFP*, resulting in no or very low green fluorescence in the infiltrated zone (**Figure 6A**). While the co-expression of known RNA silencing suppressors *p19* with *GFP* led to the recovery of green fluorescence (**Figure 6B**). Using this assay, we screened 52 candidate effectors (Supplementary Table S2; 44 genes that suppress BT-PCD, 4 genes that induce cell death and 4 genes with no obvious effect on plant defenses) of *H. avenae*, and found that no effectors suppress *GFP* silencing. As shown in **Figure 6C** (example isotig18549), *N. benthamiana* leaves infiltrated with a mixture of *A. tumefaciens* cells containing pGD-isotig18549 and pGD-sGFP showed no green fluorescence, indicating that isotig18549 did not suppress RNA silencing.

### Suppression of PTI and ETI by Candidate *H. avenae* Effectors

**FIGURE 7:**
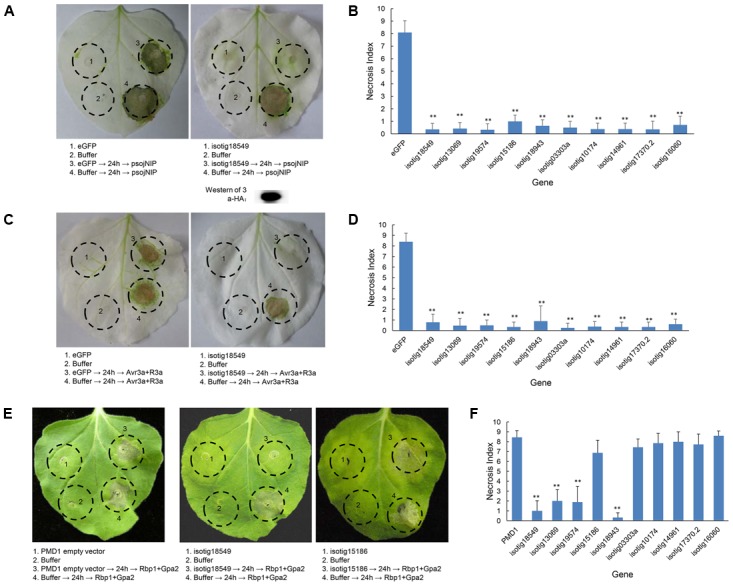
Assay of the suppression of PTI (triggered by psojNIP) and ETI (triggered by Avr3a/R3a or Rbp-1/Gpa2) by *Heterodera avenae* candidate effectors in *Nicotiana benthamiana*. **(A,C)** Visualization of the phenotype of example isotig18549, which suppressed PTI triggered by psojNIP and ETI triggered by Avr3a/R3a. Western blotting confirmed the expression of psojNIP. **(E)** Visualization of the phenotypes of necrosis suppression (example isotig18549) and no suppression (example isotig15186) of ETI triggered by Rbp-1/Gpa2). *N. benthamiana* leaves were infiltrated with buffer or with *Agrobacterium tumefaciens* cells carrying the effector genes *isotig18549* or *isotig15186* or the negative control (*eGFP* or empty vector PMD1) either alone or followed 24 h later by *A. tumefaciens* cells carrying the *psojNIP, Avr3a*/*R3a* or *Rbp-1*/*Gpa2* genes. **(B,D,F)** Necrosis indices of the infiltration spots of the 10 selected effector genes and controls (*eGFP* or empty vector PMD1) followed by infiltration with vectors carrying the *psojNIP*, *Avr3a*/*R3a* or *Rbp-1*/*Gpa2* genes. Each column shows the mean and standard deviation. The columns with asterisks show a statistically significant reduction of the necrosis index compared with the control (*P* < 0.01).

To further ascertain whether the candidate BT-PCD-suppressing effectors of *H. avenae* could suppress PTI or ETI, 10 such genes were assayed. In these experiments, psojNIP (Dinah et al., 2002) was used to trigger PTI and Avr3a/R3a (Armstrong et al., 2005) and Rbp-1/Gpa2 (Sacco et al., 2009) were used to trigger ETI by agroinfiltration. The results showed that all the 10 selected effector candidates suppressed psojNIP-induced PTI and Avr3a/R3a-induced ETI, whereas only 4 of the 10 effector candidates suppressed Rbp-1/Gpa2-induced ETI (**Table 1** and **Figures 7B,D,F**). As an example of the observed suppression, **Figure 7** shows that the infiltration spot of the candidate isotig18549 followed by each cell death inducer was not as necrotic as that of the eGFP or PMD1 empty vector control based on a quantitative comparison of the necrosis indices. The expression of psojNIP of the leaf infiltration spot of isotig18549 followed by psojNIP was verified by western blotting. The example isotig15186 failed to suppress Rbp-1/Gpa2-induced ETI (**Figure 7E**). Comparisons of the necrosis indices of all the effector candidates with those of the control are also shown (**Figures 7B,D,F**). These results indicate that CCN putative effectors may suppress plant immunity through multiple mechanisms rather than through a single pathway during parasitism.

**Table 1:** Assay of suppression of cell death triggered by BAX, psojNIP, Avr3a/R3a and Rbp-1/Gpa2 by nine selected candidate effectors of *Heterodera avenae.*

Gene ID	Hit description	BT-PCD suppression	psojNIP induced PTI suppression	Avr3a/R3a induced ETI suppression	Rbp1/Gpa2 induced ETI suppression
isotig18549	Putative gland protein G11A06 [*Heterodera glycines*]	Y	Y	Y	Y
isotig13069	Putative gland protein 30G12 [*H. glycines*]	Y	Y	Y	Y
isotig16060	14-3-3 protein [*H. glycines*]	Y	Y	Y	N
isotig15186	Calumenin-A [*Ascaris suum*]	Y	Y	Y	N
isotig18943	Putative gland protein G16B09 [*H. glycines*]	Y	Y	Y	Y
isotig19574	Transthyretin-like family protein [*Necator americanus*]	Y	Y	Y	Y
isotig14961	Putative amphid protein [*Globodera rostochiensis*]	Y	Y	Y	N
isotig10174	Disulfide-isomerase A4 [*Loa loa*]	Y	Y	Y	N
isotig17370.2	Hypothetical esophageal gland cell secretory protein 4 [*H. glycines*]	Y	Y	Y	N
isotig03303a	Ras family protein [*N. americanus*]	Y	Y	Y	N

*Y, yes; N, no*.

### Contribution of Structural Domains to PCD Induction

**FIGURE 8:**
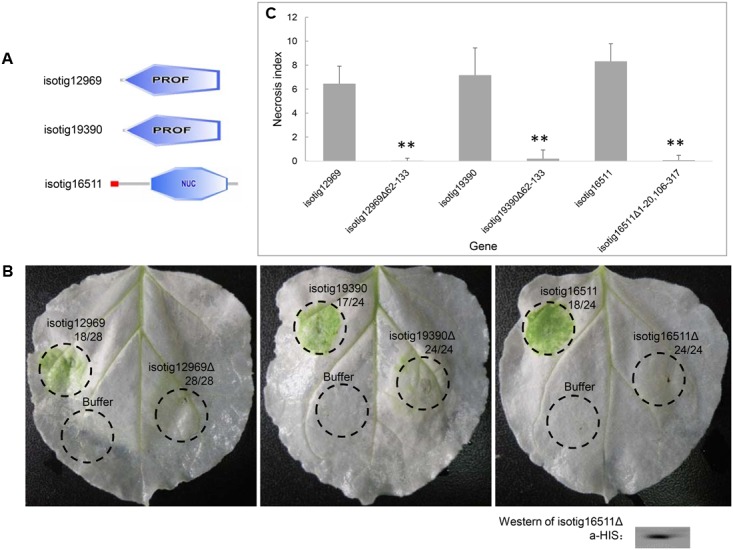
Contribution of structural domains to PCD induction by three candidate *Heterodera avenae* effectors in *Nicotiana benthamiana*. **(A)** Schematic of the structural domains of the three genes *isotig12969*, *isotig19390* and *isotig16511*. The former two genes contain PROF (profilin) domains, and the third gene encodes a SP (indicated in red) and an endonuclease domain (NUC, indicated in blue). **(B)** Visualization of the phenotypes associated with transient expression of the three genes and their respective gene fragments lacking the structural domains. The first number below the gene name indicates the total number of necrosis spots, and the second number below the gene name indicates the total number of infiltration spots. Western blotting confirmed the expression of isotig16511Δ. **(C)** Necrosis indices of the infiltration spots of the three genes and their respective gene fragments lacking the structural domains. Each column shows the mean and standard deviation. The columns with asterisks show a statistically significant reduction of the necrosis index of the gene fragments lacking the structural domains compared to those of the intact genes (*P* < 0.01).

Because we found that several candidate effector genes induced PCD in *N. benthamiana*, we further evaluated the contribution of the structural domains of their encoded proteins to PCD induction. Three of the PCD-inducing genes, *isotig12969* (hit profilin-1 [*A. suum*]), *isotig19390* (hit profilin [*B. malayi*]) and *isotig16511* (hit endonuclease G [*A. suum*]), were selected for the assay. The first two genes encode a PROF (profilin) domain (base positions 62-133), and the third gene encodes an SP domain (base positions 1–20) and an endonuclease domain (base positions 106–317) (**Figure 8A**). When the gene regions encoding these domains were deleted, the PCD induction ability of the three genes was significantly decreased (**Figures 8B,C**), indicating a determining effect of these domains in PCD induction. Western blotting verified the expression of isotig16511Δ (**Figure 8C**).

### Cooperation among *H. avenae* Candidate Effectors

**FIGURE 9:**
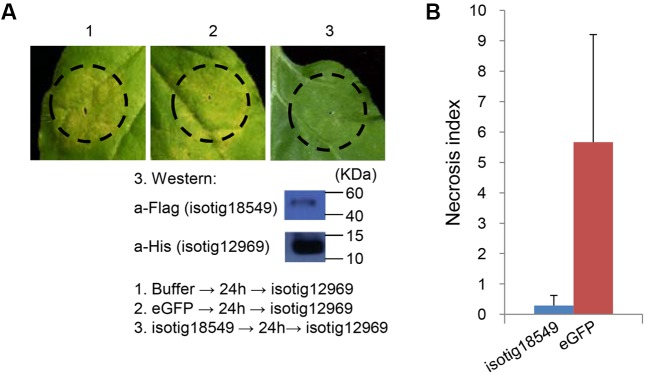
Candidate *Heterodera avenae* effectors (example isotig18549) suppress cell death triggered by other candidate *H. avenae* effectors (example isotig12969) in *Nicotiana benthamiana*. **(A)** Assay of the suppression of isotig12969-triggered cell death in *N. benthamiana* by isotig18549. The results of the verification of gene expression of isotig18549 and isotig12969 by western blotting are shown below. **(B)** Necrosis index of isotig18549 and control eGFP followed by isotig12969. Each column shows the mean and standard deviation.

As shown in the initial BT-PCD suppression assay, several CCN effector candidates could themselves trigger PCD. We therefore wondered whether the PCD triggered by these effector candidates can be suppressed by other plant-defense-suppressing CCN effectors candidates. Thus, we conducted agroinfiltration tests in *N. benthamiana.* Four genes, *isotig16511*, *isotig16978*, *isotig19390* and *isotig12969*, all of which triggered obvious cell death in *N. benthamiana* leaves, were selected as cell death inducers. Ten genes that were also selected in the PTI/ETI suppression assays listed in **Table 2** were tested for suppression ability in this experiment. As expected, candidate effector genes that suppressed cell death induced by other CCN candidate effectors did exist in the nematode (**Table 2**). For example, the infiltration spot of isotig18549 followed by the inducer isotig12969 showed little necrosis, unlike the buffer and eGFP controls followed by the inducer (**Figure 9**). This result was quantitatively confirmed by comparing the necrosis indices of isotig18549 and the eGFP control; the former was significantly smaller than the latter (**Figure 9**). With the exception of isotig16060 (hit 14-3-3 protein [*H. glycines*]), the tested BT-PCD-suppressing genes all suppressed the effects of the four inducer genes. This indicates that CCN effectors cooperate among themselves to regulate plant defenses.

**Table 2:** *Heterodera avenae* putative effectors suppress cell death triggered by other *H. avenae* putative effectors.

Genes suppressing BT-PCD	Genes inducing necrosis			
	It16511 (Endonuclease G [*Ascaris suum*])	It16978 (putative transcriptional regulator, AraC family [*Bacteroides* sp. 20_3])	It19390 (profilin [*Brugia malayi*])	It12969 (Profilin-1 [*A. suum*])
It17370.2 (hypothetical esophageal gland cell secretory protein 4 [*Heterodera glycines*])	Y	Y	Y	Y
It16060 (14-3-3 protein [*H. glycines*])	N	N	N	N
It13069 (putative gland protein 30G12 [*H. glycines*])	Y	Y	Y	Y
It19574 (Transthyretin-like family protein [*Necator americanus*])	Y	Y	Y	Y
It18549 (putative gland protein G11A06 [*H. glycines*])	Y	Y	Y	Y
It18943(putative gland protein G16B09 [*H. glycines*])	Y	Y	Y	Y
It10174 (disulfide-isomerase A4 [*Loa loa*])	Y	Y	Y	Y
It15186 (Calumenin-A [*A. suum*])	Y	Y	Y	Y
It14961 (putative amphid protein [*Globodera rostochiensis*])	Y	Y	Y	Y
It03303a (Ras family protein [*N. americanus*])	Y	Y	Y	Y

*It, isotig; Y, yes; N, no*.

## Discussion

The effector proteins secreted by PPNs play essential roles in host–pathogen interactions (Mitchum et al., 2013). Transient expression assays in the *N. benthamiana* model system employing agroinfiltration have been successfully used to identify many bacterial, oomycete, fungal and nematode effectors (Guo et al., 2009; Wang et al., 2011; Zhang et al., 2014; Ali et al., 2015). In this study, we cloned 95 putative effectors from an effector repertoire identified by bioinformatics analysis of the *H. avenae* transcriptome. We found that 78 putative effectors suppressed PCD triggered by BAX and 7 putative effectors induced cell death or chlorosis in *N. benthamiana* (**Figure 1A** and Supplementary Table S2). The two-step Agro-infiltration protocol may result in that the prior infiltration inhibit subsequent T-DNA transfer, but the level of BAX protein was identical in tissues showing suppression and those not showing suppression (Wang et al., 2011). So the suppression of BT-PCD did not result from suppression of BAX DNA delivery or PVX massive replication after the second *A. tumefaciens* infiltration. In addition, although our assays were conducted in the non-host *N. benthamiana*, many studies of other pathogen effectors have shown that these effectors possess the same ability to suppress or induce cell death in non-host and host plants. For example, Avh172 and Avh6 of *P. sojae* were shown to suppress ETI in both the non-host *N. benthamiana* and in host soybean (Wang et al., 2011). Of the five *M. oryzae* cell-death-inducing proteins, all induce cell death in both host rice and non-host maize protoplasts, and four also cause cell death in the protoplasts of the dicot plants *A. thaliana* and *N. benthamiana* (Chen et al., 2013). These results demonstrate that the plant defenses machinery is well-conserved in diverse plant families. Accordingly, the transient expression assay in *N. benthamiana* is an efficient method for preliminary large-scale screening of putative effectors that may suppress or induce cell death. Further investigations (such as ROS assay, callose deposition, immunization-related gene expression. etc.) will be performed to verify the plant immunity suppressing abilities of the putative effectors.

To ascertain the characteristics such as the secretion ability and the role in parasitism of the selected candidate effectors, experimental confirmation was conducted. The yeast secretion assay has been used to investigate the secretion of predicted effectors (Chen et al., 2013; Fang et al., 2016). Using this assay, we verified that 33 of 53 predicted SPs of *H. avenae* putative effectors were functional in the yeast secretion system (Supplementary Table S2). Furthermore, the developmental expression pattern results demonstrated that most of the 30 tested effector candidates of *H. avenae* were transcriptionally expressed at higher levels during parasitic stages than during pre-parasitic stages (**Figure 2**), indicating that these putative effectors play important roles in parasitism. In addition, the ten tested effectors were all observed in gland cells of *H. avenae* by *in situ* hybridization (**Figure 3**); thus, they display a major feature of nematode effector proteins. Previous research has shown that systemic expression of effectors *in planta* is an effective method that can be used to identify effectors that cause dramatic phenotypes in plants (Ali et al., 2015). In our work, 56 of 67 tested effector candidates induced symptoms during systemic expression in *N. benthamiana*; these symptoms included necrosis, wilting, dwarfing and aggravation of PVX symptoms (**Figure 5** and Supplementary Table S2), demonstrating that the putative effectors may play some roles in plants.

Recently, several suppressors of RNA silencing, a common counter-defense strategy used by viruses, have been reported among bacterial and oomycete pathogens (Navarro et al., 2008; Qiao et al., 2013). Suppression of the RNA silencing pathway is observed during RKN infection of *N. tabacum*, but no specific suppressors of RNA silencing have been identified (Walsh et al., 2017). We attempted to find suppressors of RNA silencing in *H. avenae* by agroinfiltration of *N. benthamiana*. However, when the 52 tested candidate effectors of *H. avenae* were co-infiltrated with eGFP, we observed no suppression of RNA silencing (Supplementary Table S2). It would probably be more useful to screen more effector candidates for silencing suppressor activities.

PTI and ETI comprise two branches of plant defenses. Ten BT-PCD-suppressing effector candidates in *H. avenae* were randomly selected, and their ability to suppress PTI or ETI was tested. As expected, all of the selected effector candidates could also suppress PCD triggered by the elicitor PsojNIP (**Figure 7B**) and at least one R-protein/cognate effector pair (**Figures 7D,F**). PsojNIP acts as an elicitor in a manner similar to PAMPs, which induce necrosis at the extracytoplasmic side of the host plasma membrane (Qutob et al., 2006). The resistance proteins R3a and Gpa2 recognize their respective elicitors, Avr3a and RBP-1, intracellularly to induce ETI (Huang et al., 2005; Sacco et al., 2009). Our results indicated that all the tested putative effectors of *H. avenae* could suppress both PTI and ETI (**Table 1**), and they were active in suppressing cell death not only in apoplasts but also in the cytoplasm. The high proportion of putative effectors that were found to suppress PCD suggests that suppression of plant immunity is one of the primary ways in which *H. avenae* effectors contribute to biotrophic parasitism. Our results are consistent with previous findings in *P. syringae* and *P. sojae* that most tested effectors can suppress plant defenses (Guo et al., 2009; Wang et al., 2011).

Biotrophic pathogens usually avoid triggering plant cell death; instead, they typically suppress plant defenses for survival. Surprisingly, 7 of 95 putative effectors in *H. avenae* were shown to have the ability to induce cell death in *N. benthamiana* (**Figure 1B**), indicating that these proteins might be recognized by the plant defenses machinery or might function as toxins to induce necrosis. For hemibiotrophic plant pathogens, which undergo a necrotrophic phase following the biotrophic phase, some effectors in *P. sojae* and *M. oryzae* have been shown to induce cell death (Wang et al., 2011; Chen et al., 2013). These cell-death-inducing effectors may facilitate the colonization of pathogens during the late necrotrophic phase of infection. To date, in nematodes, only expansin and expansin-like proteins (GrEXPB2, HaEXPB1 and HaEXPB2) have been reported to act alone to trigger cell death in plants (Ali et al., 2015; Liu et al., 2016). These proteins fail to induce cell death when their SPs are deleted, indicating that they only function when present in the apoplastic space of the plant cell. Expansins located outside the cell may bind to and alter the plant cell wall, resulting in cell death (Liu et al., 2016). In contrast, in our study, the 7 cell-death-inducing putative effectors all functioned in the absence of SPs. Therefore, these putative effectors appear to trigger cell death by acting within the cytoplasm of the plant cell. However, the functions of the cell-death-inducing effectors in biotrophic *H. avenae* remain to be elucidated.

Interestingly, cooperation between effectors that suppress PCD and those that induce PCD was observed in *H. avenae*. Of the ten putative effectors that suppressed BT-PCD, nine also suppressed PCD triggered by the cell-death-inducing putative effectors themselves (**Table 2**). This result is consistent with the hypothesis that biotrophic nematodes must avoid inducing host defenses for successful parasitism. In *G. pallida*, the effector RBP-1 is recognized by the potato resistance protein Gpa2 and elicits HR, whereas two SPRYSECs of *G. pallida* can suppress the induced cell death (Mei et al., 2015). Cooperation between effectors was also reported in *P. sojae*, in which it was shown that all of the effectors that suppress BT-PCD could also suppress PCD triggered by at least one effector (Avh238 and Avh241) (Wang et al., 2011). Although the induction of cell death is related to the triggering of plant defenses mechanisms, it has been reported that cell death induction might not contribute to host resistance. HaEXPB2 induces cell death, but treatment with RNAi for HaEXPB2 does not significantly change the number of cysts produced in the host (Liu et al., 2016), suggesting that HaEXPB2 has no effect on host resistance. Similarly, we presumed that some cell-death-inducing effectors of *H. avenae* might be recognized by plants and induce defense responses, whereas other effectors cooperate in suppressing the induced defense responses. Through this cooperation, *H. avenae* ensures that the recognition of cell-death-inducing effectors does not confer resistance in plants.

Conserved domains are reported to be essential for the function of cell-death-inducing effectors (Chen et al., 2013; Fang et al., 2016). The entire HaEXPB2 protein, including the C-terminus, the β-expansin domain, and the carbohydrate-binding domain, is necessary for inducing cell death in *N. benthamiana* (Liu et al., 2016). Through a Pfam search, isotig12969 and isotig19390 were each predicted to contain a PROF (profilin) domain, which is typically involved in actin binding. Actin reorganization is crucial for the development and expansion of nematode feeding sites. When Mi131, a protein from *M. incognita* that bears a profilin domain, was expressed in protoplasts in which the actin cytoskeleton had been labeled with GFP, the actin cytoskeleton appeared fragmented (Leelarasamee, 2015). Profilin from the protozoan parasite *Toxoplasma gondii* plays a role in motility and serves as a microbial ligand that is recognized by the host innate immune system, functioning like bacterial flagellin (Plattner et al., 2008). In our study, isotig12969 and isotig19390 lacking the PROF domain lost the ability to induce cell death (**Figure 8**); this result is consistent with the above reports that profilin resembles PAMPs. Isotig16511 was predicted to be an endonuclease G, a type of endonuclease that acts as a PCD DNase when released from mitochondria (Li et al., 2001). As expected, isotig16511 lacking a NUC domain was unable to trigger cell death (**Figure 8**), consistent with the predicted function of this protein.

Although the tested *H. avenae* putative effectors in this study has been nearly one hundreds, it will be necessary to examine more effectors in the future. Our study provides a method for the large-scale and rapid investigation of the effector repertoire of PPN in the suppression of plant defenses mechanisms. Our data show that the majority of the effectors predicted based on transcriptomic analysis have the potential to suppress plant defenses, including PTI and ETI. This study reveals the important role of *H. avenae* effectors in biotrophic infection and significantly advances our understanding of the defense-suppressing function of PPN effectors.

## Author Contributions

HJ and QL conceived the idea, acquired funding and designed the experiments. CC, YC, LP, YD, FS, and SY performed the experiments. CC and DY contributed toward the data analyses. CC, QL, and HJ wrote and revised the manuscript. All authors read and approved the final version of the manuscript for publication.

## Conflict of Interest Statement

The authors declare that the research was conducted in the absence of any commercial or financial relationships that could be construed as a potential conflict of interest.

## Footnotes

1 https://www.biocloud.net/

2 http://smart.embl-heidelberg.de/

3 https://www.biocloud.net/

## References

[B1] Abad P. Gouzy J. Aury J. Castagnone-Sereno P. Danchin E. G. J. Deleury E. (2008). Genome sequence of the metazoan plant-parasitic nematode *Meloidogyne incognita*. *Nat. Biotechnol.* 26 909–915. 10.1038/nbt.1482 18660804

[B2] Abramovitch R. B. Kim Y. J. Chen S. R. Dickman M. B. Martin G. B. (2003). *Pseudomonas* type III effector AvrPtoB induces plant disease susceptibility by inhibition of host programmed cell death. *EMBO J.* 22 60–69. 10.1093/emboj/cdg006 12505984 PMC140047

[B3] Ali S. Magne M. Chen S. Cote O. Stare B. G. Obradovic N. (2015). Analysis of putative apoplastic effectors from the nematode, *Globodera rostochiensis*, and identification of an expansin-like protein that can induce and suppress host defenses. *PLOS ONE* 10:e0115042. 10.1371/journal.pone.0115042 25606855 PMC4301866

[B4] Armstrong M. R. Whisson S. C. Pritchard L. Bos J. I. Venter E. Avrova A. O. (2005). An ancestral oomycete locus contains late blight avirulence gene Avr3a, encoding a protein that is recognized in the host cytoplasm. *Proc. Natl. Acad. Sci. U.S.A.* 102 7766–7771. 10.1073/pnas.0500113102 15894622 PMC1140420

[B5] Boller T. He S. Y. (2009). Innate immunity in plants: an arms race between pattern recognition receptors in plants and effectors in microbial pathogens. *Science* 324 742–744. 10.1126/science.1171647 19423812 PMC2729760

[B6] Bonfil D. J. Dolgin B. Mufradi I. Asido S. (2004). Bioassay to forecast cereal cyst nematode damage to wheat in fields. *Precis. Agric.* 5 329–344. 10.1023/B:PRAG.0000040804.97462.02

[B7] Chen C. Liu S. Liu Q. Niu J. Liu P. Zhao J. (2015). An ANNEXIN-like protein from the cereal cyst nematode *Heterodera avenae* suppresses plant defense. *PLOS ONE* 10:e0122256. 10.1371/journal.pone.0122256 25849616 PMC4388550

[B8] Chen S. Songkumarn P. Venu R. C. Gowda M. Bellizzi M. Hu J. (2013). Identification and characterization of in planta-expressed secreted effector proteins from *Magnaporthe oryzae* that induce cell death in rice. *Mol. Plant Microbe Interact.* 26 191–202. 10.1094/MPMI-05-12-0117-R 23035914

[B9] Chisholm S. T. Coaker G. Day B. Staskawicz B. J. (2006). Host-microbe interactions: shaping the evolution of the plant immune response. *Cell* 124 803–814. 10.1016/j.cell.2006.02.008 16497589

[B10] Chronis D. Chen S. Lu S. Hewezi T. Carpenter S. C. D. Loria R. (2013). A ubiquitin carboxyl extension protein secreted from a plant-parasitic nematode *Globodera rostochiensis* is cleaved in planta to promote plant parasitism. *Plant J.* 74 185–196. 10.1111/tpj.12125 23346875

[B11] Diaz-Granados A. Petrescu A. Goverse A. Smant G. (2016). SPRYSEC effectors: a versatile protein-binding platform to disrupt plant innate immunity. *Front. Plant Sci.* 7:1575. 10.3389/fpls.2015.01575 27812363 PMC5071358

[B12] Dinah Q. Sophien K. Mark G. (2002). Expression of a *Phytophthora sojae* necrosis-inducing protein occurs during transition from biotrophy to necrotrophy. *Plant J. Cell Mol. Biol.* 32 361–373. 12410814 10.1046/j.1365-313x.2002.01439.x

[B13] Ding S. W. (2010). RNA-based antiviral immunity. *Nat. Rev. Immunol.* 10 632–644. 10.1038/nri2824 20706278

[B14] Dou D. Kale S. D. Wang X. Chen Y. Wang Q. Wang X. (2008). Conserved C-terminal motifs required for avirulence and suppression of cell death by Phytophthora sojae effector Avr1b. *Plant Cell* 20 1118–1133. 10.1105/tpc.107.057067 18390593 PMC2390733

[B15] Eves-van den Akker S. Laetsch D. R. Thorpe P. Lilley C. J. Danchin E. G. J. Rocha M. D. (2016). The genome of the yellow potato cyst nematode, *Globodera rostochiensis*, reveals insights into the basis of parasitism and virulence. *Genome Biol.* 17 124. 10.1186/s13059-016-0985-1 27286965 PMC4901422

[B16] Fang A. Han Y. Zhang N. Zhang M. Liu L. Li S. (2016). Identification and characterization of plant cell death-inducing secreted proteins from *Ustilaginoidea virens*. *Mol. Plant Microbe Interact.* 29 405–416. 10.1094/MPMI-09-15-0200-R 26927000

[B17] Favery B. Quentin M. Jaubert-Possamai S. Abad P. (2016). Gall-forming root-knot nematodes hijack key plant cellular functions to induce multinucleate and hypertrophied feeding cells. *J. Insect Physiol.* 84 60–69. 10.1016/j.jinsphys.2015.07.013 26211599

[B18] Gleason C. A. Liu Q. L. Williamson V. M. (2008). Silencing a candidate nematode effector gene corresponding to the tomato resistance gene Mi-1 leads to acquisition of virulence. *Mol. Plant Microbe Interact.* 21 576–585. 10.1094/MPMI-21-5-0576 18393617

[B19] Goodin M. M. Dietzgen R. G. Schichnes D. Ruzin S. Jackson A. O. (2002). pGD vectors: versatile tools for the expression of green and red fluorescent protein fusions in agroinfiltrated plant leaves. *Plant J.* 31 375–383. 10.1046/j.1365-313X.2002.01360.x 12164816

[B20] Gu B. Kale S. D. Wang Q. Wang D. Pan Q. Cao H. (2011). Rust secreted protein Ps87 is conserved in diverse fungal pathogens and contains a RXLR-like motif sufficient for translocation into plant cells. *PLOS ONE* 6:e27217. 10.1371/journal.pone.0027217 22076138 PMC3208592

[B21] Guo M. Tian F. Wamboldt Y. Alfano J. R. (2009). The Majority of the type III effector inventory of *Pseudomonas syringae* pv. tomato DC3000 can suppress plant immunity. *Mol. Plant Microbe Interact.* 22 1069–1080. 10.1094/MPMI-22-9-1069 19656042 PMC2778199

[B22] Haegeman A. Mantelin S. Jones J. T. Gheysen G. (2012). Functional roles of effectors of plant-parasitic nematodes. *Gene* 492 19–31. 10.1016/j.gene.2011.10.040 22062000

[B23] Hogenhout S. A. Van der Hoorn R. A. L. Terauchi R. Kamoun S. (2009). Emerging concepts in effector biology of plant-associated organisms. *Mol. Plant Microbe Interact.* 22 115–122. 10.1094/MPMI-22-2-0115 19132864

[B24] Huang G. Gao B. Maier T. Allen R. Davis E. L. Baum T. J. (2003). A profile of putative parasitism genes expressed in the esophageal gland cells of the root-knot nematode *Meloidogyne incognita*. *Mol. Plant Microbe Interact.* 16 376–381. 10.1094/MPMI.2003.16.5.376 12744507

[B25] Huang S. W. van der Vossen E. Kuang H. H. Vleeshouwers V. Zhang N. W. Borm T. (2005). Comparative genomics enabled the isolation of the R3a late blight resistance gene in potato. *Plant J.* 42 251–261. 10.1111/j.1365-313X.2005.02365.x 15807786

[B26] Iberkleid I. Vieira P. Engler J. D. A. Firester K. Spiegel Y. Horowitz S. B. (2013). Fatty acid-and retinol-binding protein, Mj-FAR-1 induces tomato host susceptibility to root-knot nematodes. *PLOS ONE* 8:e64586. 10.1371/journal.pone.0064586 23717636 PMC3661543

[B27] Incarbone M. Dunoyer P. (2013). RNA silencing and its suppression: novel insights from in planta analyses. *Trends Plant Sci.* 18 382–392. 10.1016/j.tplants.2013.04.001 23684690

[B28] Jacobs K. A. CollinsRacie L. A. Colbert M. Duckett M. GoldenFleet M. Kelleher K. (1997). A genetic selection for isolating cDNAs encoding secreted proteins. *Gene* 198 289–296. 10.1016/S0378-1119(97)00330-2 9370294

[B29] Jaouannet M. Magliano M. Arguel M. J. Gourgues M. Evangelisti E. Abad P. (2013). The root-knot nematode calreticulin Mi-CRT is a key effector in plant defense suppression. *Mol. Plant Microbe Interact.* 26 97–105. 10.1094/MPMI-05-12-0130-R 22857385

[B30] Jones J. D. G. Dangl J. L. (2006). The plant immune system. *Nature* 444 323–329. 10.1038/nature05286 17108957

[B31] Jones L. Hamilton A. J. Voinnet O. Thomas C. L. Maule A. J. Baulcombe D. C. (1999). RNA-DNA interactions and DNA methylation in post-transcriptional gene silencing. *Plant Cell* 11 2291–2301. 10.1105/tpc.11.12.2291 10590159 PMC144133

[B32] Kumar M. Gantasala N. P. Roychowdhury T. Thakur P. K. Banakar P. Shukla R. N. (2014). De novo transcriptome sequencing and analysis of the cereal cyst nematode, *Heterodera avenae*. *PLOS ONE* 9:e96311. 10.1371/journal.pone.0096311 24802510 PMC4011697

[B33] Leelarasamee N. (2015). *The Functional Characterization of a Root Knot Nematode Effector Mi131.* Ph.D. dissertation, University of Göttingen, Göttingen.

[B34] Li L. Y. Luo L. Wang X. D. (2001). Endonuclease G is an apoptotic DNase when released from mitochondria. *Nature* 412 95–99. 10.1038/35083620 11452314

[B35] Li X. Yang D. Niu J. Zhao J. Jian H. (2016). De novo analysis of the transcriptome of *Meloidogyne enterolobii* to uncover potential target genes for biological control. *Int. J. Mol. Sci.* 17:E1442. 10.3390/ijms17091442 27598122 PMC5037721

[B36] Lin B. Zhuo K. Chen S. Hu L. Sun L. Wang X. (2016). A novel nematode effector suppresses plant immunity by activating host reactive oxygen species-scavenging system. *New Phytol.* 209 1159–1173. 10.1111/nph.13701 26484653 PMC5057313

[B37] Liu J. Peng H. Cui J. Huang W. Kong L. Clarke J. L. (2016). Molecular characterization of A novel effector expansin-like protein from *Heterodera avenae* that induces cell death in *Nicotiana benthamiana*. *Sci. Rep.* 6:35677. 10.1038/srep35677 27808156 PMC5093861

[B38] Lozano-Torres J. L. Wilbers R. H. P. Gawronski P. Boshoven J. C. Finkers-Tomczak A. Cordewener J. H. G. (2012). Dual disease resistance mediated by the immune receptor Cf-2 in tomato requires a common virulence target of a fungus and a nematode. *Proc. Natl. Acad. Sci. U.S.A.* 109 10119–10124. 10.1073/pnas.1202867109 22675118 PMC3382537

[B39] Lozano-Torres J. L. Wilbers R. H. P. Warmerdam S. Finkers-Tomczak A. Diaz-Granados A. van Schaik C. C. (2014). Apoplastic venom allergen-like proteins of cyst nematodes modulate the activation of basal plant innate immunity by cell surface receptors. *PLOS Pathog.* 10:e1004569. 10.1371/journal.ppat.1004569 25500833 PMC4263768

[B40] Mei Y. Thorpe P. Guzha A. Haegeman A. Blok V. C. MacKenzie K. (2015). Only a small subset of the SPRY domain gene family in *Globodera pallida* is likely to encode effectors, two of which suppress host defences induced by the potato resistance gene Gpa2. *Nematology* 17 409–424. 10.1163/15685411-00002875

[B41] Mitchum M. G. Hussey R. S. Baum T. J. Wang X. Elling A. A. Wubben M. (2013). Nematode effector proteins: an emerging paradigm of parasitism. *New Phytol.* 199 879–894. 10.1111/nph.12323 23691972

[B42] Navarro L. Jay F. Nomura K. He S. Y. Voinnet O. (2008). Suppression of the microRNA pathway by bacterial effector proteins. *Science* 321 964–967. 10.1126/science.1159505 18703740 PMC2570098

[B43] Nicol J. M. Elekcioglu I. H. Bolat N. Rivoal R. (2007). The global importance of the cereal cyst nematode (*Heterodera* spp.) on wheat and international approaches to its control. *Commun. Agric. Appl. Biol. Sci.* 72 677–686. 18399504

[B44] Niu J. Liu P. Liu Q. Chen C. Guo Q. Yin J. (2016). Msp40 effector of root-knot nematode manipulates plant immunity to facilitate parasitism. *Sci. Rep.* 6:19443. 10.1038/srep19443 26797310 PMC4726423

[B45] Noon J. B. Qi M. Sill D. N. Muppirala U. Eves-van Den Akker S. Maier T. R. (2016). A Plasmodium-like virulence effector of the soybean cyst nematode suppresses plant innate immunity. *New Phytol.* 212 444–460. 10.1111/nph.14047 27265684

[B46] Oh S. K. Young C. Lee M. Oliva R. Bozkurt T. O. Cano L. M. (2009). In planta expression screens of *Phytophthora infestans* RXLR effectors reveal diverse phenotypes, including activation of the *Solanum bulbocastanum* disease resistance protein Rpi-blb2. *Plant Cell Online* 21 2928–2947. 10.1105/tpc.109.068247 19794118 PMC2768934

[B47] Opperman C. H. Bird D. M. Williamson V. M. Rokhsar D. S. Burke M. Cohn J. (2008). Sequence and genetic map of *Meloidogyne hapla*: a compact nematode genome for plant parasitism. *Proc. Natl. Acad. Sci. U.S.A.* 105 14802–14807. 10.1073/pnas.0805946105 18809916 PMC2547418

[B48] Plattner F. Yarovinsky F. Romero S. Didry D. Carlier M. Sher A. (2008). Toxoplasma profilin is essential for host cell invasion and TLR11-dependent induction of an interleukin-12 response. *Cell Host Microbe* 3 77–87. 10.1016/j.chom.2008.01.001 18312842

[B49] Qiao Y. Liu L. Xiong Q. Flores C. Wong J. Shi J. (2013). Oomycete pathogens encode RNA silencing suppressors. *Nat. Genet.* 45 330–333. 10.1038/ng.2525 23377181 PMC4049077

[B50] Qutob D. Kemmerling B. Brunner F. Kuefner I. Engelhardt S. Gust A. A. (2006). Phytotoxicity and innate immune responses induced by Nep1-like proteins. *Plant Cell* 18 3721–3744. 10.1105/tpc.106.044180 17194768 PMC1785393

[B51] Sacco M. A. Koropacka K. Grenier E. Jaubert M. J. Blanchard A. Goverse A. (2009). The cyst nematode SPRYSEC protein RBP-1 elicits Gpa2-and RanGAP2-dependent plant cell death. *PLOS Pathog.* 5:e1000564. 10.1371/journal.ppat.1000564 19714238 PMC2727447

[B52] Sobezak M. Golinowski W. (2009). “Structure of cyst nematode feeding sites,” in *Plant Cell Monographs*, eds Berg R. H. Taylor C. G. (Berlin: Springer), 153–187.

[B53] Tai T. H. Dahlbeck D. Clark E. T. Gajiwala P. Pasion R. Whalen M. C. (1999). Expression of the Bs2 pepper gene confers resistance to bacterial spot disease in tomato. *Proc. Natl. Acad. Sci. U.S.A.* 96 14153–14158. 10.1073/pnas.96.24.14153 10570214 PMC24206

[B54] Thorpe P. Mantelin S. Cock P. J. Blok V. C. Coke M. C. Eves-van Den Akker S. (2014). Genomic characterisation of the effector complement of the potato cyst nematode *Globodera pallida*. *BMC Genomics* 15:923. 10.1186/1471-2164-15-923 25342461 PMC4213498

[B55] Tsuda K. Katagiri F. (2010). Comparing signaling mechanisms engaged in pattern-triggered and effector-triggered immunity. *Curr. Opin. Plant Biol.* 13 459–465. 10.1016/j.pbi.2010.04.006 20471306

[B56] Walsh E. Elmore J. M. Taylor C. G. (2017). Root-knot nematode parasitism suppresses host RNA silencing. *Mol. Plant Microbe Interact.* 30 295–300. 10.1094/MPMI-08-16-0160-R 28402184

[B57] Wang Q. Han C. Ferreira A. O. Yu X. Ye W. Tripathy S. (2011). Transcriptional programming and functional interactions within the *Phytophthora sojae* RXLR effector repertoire. *Plant Cell* 23 2064–2086. 10.1105/tpc.111.086082 21653195 PMC3160037

[B58] Xiong Q. Ye W. Choi D. Wong J. Qiao Y. Tao K. (2014). *Phytophthora* suppressor of RNA silencing 2 is a conserved RxLR effector that promotes infection in soybean and *Arabidopsis thaliana*. *Mol. Plant Microbe Interact.* 27 1379–1389. 10.1094/MPMI-06-14-0190-R 25387135

[B59] Yang D. Chen C. Liu Q. Jian H. (2017). Comparative analysis of pre- and post-parasitic transcriptomes and mining pioneer effectors of *Heterodera avenae*. *Cell Biosci.* 7 11. 10.1186/s13578-017-0138-6 28289537 PMC5309974

[B60] Zhang L. D. Wang Z. H. Wang X. B. Li D. W. Han C. G. Zhai Y. F. (2005). Two virus-encoded RNA silencing suppressors, P14 of beet necrotic yellow vein virus and S6 of Rice black streak dwarf virus. *Chin. Sci. Bull.* 50 305–310. 10.1360/982004-731

[B61] Zhang X. Liu D. Yan T. Fang X. Dong K. Xu J. (2017). Cucumber mosaic virus coat protein modulates the accumulation of 2b protein and antiviral silencing that causes symptom recovery in planta. *PLOS Pathog.* 13:e1006522. 10.1371/journal.ppat.1006522 28727810 PMC5538744

[B62] Zhang Y. Zhang K. Fang A. Han Y. Yang J. Xue M. (2014). Specific adaptation of *Ustilaginoidea virens* in occupying host florets revealed by comparative and functional genomics. *Nat. Commun.* 5:3849. 10.1038/ncomms4849 24846013

[B63] Zheng M. Long H. Zhao Y. Li L. Xu D. Zhang H. (2015). RNA-Seq based identification of candidate parasitism genes of cereal cyst nematode (*Heterodera avenae*) during incompatible infection to *Aegilops variabilis*. *PLOS ONE* 10:e0141095. 10.1371/journal.pone.0141095 26517841 PMC4627824

